# Research on the Impacts of Cognitive Style and Computational Thinking on College Students in a Visual Artificial Intelligence Course

**DOI:** 10.3389/fpsyg.2022.864416

**Published:** 2022-05-26

**Authors:** Chi-Jane Wang, Hua-Xu Zhong, Po-Sheng Chiu, Jui-Hung Chang, Pei-Hsuan Wu

**Affiliations:** ^1^Department of Nursing, College of Medicine, National Cheng Kung University, Tainan, Taiwan; ^2^Department of Engineering Science, National Cheng Kung University, Tainan, Taiwan; ^3^Department of E-Learning Design and Management, National Chiayi University, Chiayi, Taiwan; ^4^Computer and Network Center, and Department of Computer Science and Information Engineering, National Cheng Kung University, Tainan, Taiwan; ^5^Department of Computer Science and Information Engineering, National Cheng Kung University, Tainan, Taiwan

**Keywords:** artificial intelligence, cognitive style, computational thinking, visual programming language, higher education

## Abstract

Visual programming language is a crucial part of learning programming. On this basis, it is essential to use visual programming to lower the learning threshold for students to learn about artificial intelligence (AI) to meet current demands in higher education. Therefore, a 3-h AI course with an RGB-to-HSL learning task was implemented; the results of which were used to analyze university students from two different disciplines. Valid data were collected for 65 students (55 men, 10 women) in the Science (Sci)-student group and 39 students (20 men, 19 women) in the Humanities (Hum)-student group. Independent sample *t*-tests were conducted to analyze the difference between cognitive styles and computational thinking. No significant differences in either cognitive style or computational thinking ability were found after the AI course, indicating that taking visual AI courses lowers the learning threshold for students and makes it possible for them to take more difficult AI courses, which in turn effectively helping them acquire AI knowledge, which is crucial for cultivating talent in the field of AI.

## Introduction

Increases in the use of artificial intelligence (AI) in education provide teachers with more practical guidance functions and new ways of teaching (Zhang and Aslan, 2021). The rapid rise in the use of AI has created many challenges and problems in higher education, and teachers, thus, must rethink their pedagogy and role in the use of AI technology to enhance student learning (Popenici and Kerr, 2017). AI is more difficult to learn than programming because it requires students to have a specific level of programming skills and complex knowledge. These challenges make it difficult to extend the learning of AI to higher education. Furthermore, the implementation of visual programming (e.g., Scratch) at higher education levels is no longer appropriate for students with specific programming skills and high levels of knowledge (Hu et al., 2021). First-time students encounter a variety of programming difficulties and challenges that make it difficult for them to develop programming skills (Yukselturk and Altiok, 2017). In the face of difficult learning challenges in traditional programming environments, it is vital for teachers to use visual programming environments to help students learn programming (Lye and Koh, 2014; Yukselturk and Altiok, 2017). Likewise, the use of a visual programming block approach in the AI curriculum can help students understand programming concepts and reduce misunderstandings.

In response to the current demand for AI learning and visual programming in higher education, in this study, an AI curriculum intended to address the current challenges of AI learning in higher education is implemented. Some studies have shown that the implementation of AI courses can enhance specific aspects of learning (e.g., cognitive abilities, skills) (Chai et al., 2021; Huang, 2021; Kong et al., 2021). This AI course uses an AI platform with a visual interface to increase students’ understanding of AI and lower the threshold and pressure related to machine learning. However, some studies have shown that humanities and science students have different learning abilities in different subjects (Billington et al., 2007; Katai, 2020). It is important to investigate the differences between students’ prior knowledge and cognitive styles to implement an AI curriculum. In particular, Kong et al. (2021) indicated that prior knowledge is not an important consideration in developing AI literacy in students. In summary, implementation of an AI curriculum requires an understanding of the impact of different aspects of student learning (e.g., cognitive style, computational thinking), which is crucial to improving the value of feedback related to the AI curriculum.

Cognitive style is often defined as different ways in which individuals process information (Papanikolaou et al., 2006). There are three different cognitive styles: creative, knowing, and planning (Cools and Van den Broeck, 2007). Cognitive style is an important learning factor because students have different learning preferences and adopt different learning strategies (Papanikolaou et al., 2006; Chen, 2010; Bouckenooghe et al., 2016). The learning strategies adopted by students are very important to their learning situation and learning needs (Rodrigues et al., 2019). Students in different educational departments will adopt different learning strategies and will, in turn, have different learning needs. It is necessary to understand what cognitive styles students use to solve problems in AI courses. In particular, students with different cognitive styles may have different computational thinking skills. A deeper understanding of students’ cognitive styles can provide teachers with information that will help enhance learning outcomes.

Since Wing (2006) promoted computational thinking in education and made it popular, and many teachers have begun to incorporate computational thinking into their courses. A common computational thinking problem-solving process has five basic elements (abstraction, decomposition, algorithmic thinking, evaluation, and generalization) (Selby and Woollard, 2013; Anderson, 2016; Tsai et al., 2021). It is crucial for teachers to use technology-based teaching and assessment tools to develop computational thinking skills in their students. For example, Tsai et al. (2021) developed a computational thinking assessment tool to assess students’ learning preferences and habits in the subject. Students enrolled in AI courses do not necessarily have extensive programming experience and have to learn mathematical and programming concepts in an easily explained manner (Kolachalama and Garg, 2018). Visual programming blocks may provide a convenient way for teachers to teach computational thinking, thus developing students’ computational thinking skills (Lye and Koh, 2014; Hsu et al., 2018; Xu et al., 2019). Teaching tools that reflect students’ learning levels are essential (Wong et al., 2020) and can help students understand AI concepts and acquire computational thinking skills through visual programming blocks (Lye and Koh, 2014). To help students face difficult AI courses, it is important to use visual programming blocks that will help them become familiar with AI skills and concepts.

However, there is no relevant research on implementation of a visual AI curriculum, and further exploration of students’ computational thinking skills and their cognitive styles is needed. This study implements an AI course that provides students with the ability to learn an AI course content and to share their models to enhance their computational thinking skills. Kong et al. (2021) indicated that the implementation of an AI course can enhance the acquisition of AI concepts among students at all levels and can reduce gaps for students with different educational backgrounds and different skill levels. Therefore, university students in different disciplines from two different universities served as participants in this study, and a visual AI platform was used to conduct an AI course. A teaching experiment was conducted to analyze the cognitive styles and computational thinking skills of university students from two different academic departments. The study was aimed at understanding the cognitive styles and computational thinking skills of the students from different departments in the AI course. Therefore, the following research questions were proposed in this study:

1. Does an AI visual programming course have different impacts on cognitive style among students from different departments?

2. Does an AI visual programming course have different impacts on computational thinking skills among students from different departments?

## Literature Review

### Visual Programming Learning Environment

Visual programming plays an important role in programming education to promote students’ understanding of programming and to maximize their engagement in problem-solving (Mladenović et al., 2021). Visual programming provides a programming interface that helps students learn programming concepts and processes (Lye and Koh, 2014; Chao, 2016; Essel et al., 2017; Mladenović et al., 2018; Scherer et al., 2020). In particular, Hu et al. (2021) indicated that visual programming can improve student’s academic performance with a small to medium significant overall mean effect size in this area. A visual programming environment allows students to focus on developing and designing programs (Mladenović et al., 2018; Topalli and Cagiltay, 2018), so they become more motivated to solve programming problems without grammar constraints. Visual programming has major learning benefits for students, including an intuitive programming interface and reduced difficulties related to programming (Lye and Koh, 2014; Essel et al., 2017; Mladenović et al., 2018; Lindberg et al., 2019). Considering the need to effectively develop AI talents at the university level, it is necessary to consider the learning benefits provided by a visual programming learning environment. It helps university students quickly learn about AI and helps them gain a better understanding of AI.

### Artificial Intelligence Courses

Artificial Intelligence (AI) is a scientific discipline based on algorithms intended to lead to an understanding of real human environments and construction of solutions (Schinkel et al., 2019). In terms of learning in the field of AI, machine learning courses are a learning opportunity to advance into the field of AI (Jiang and Li, 2021). Some studies have implemented AI courses for university students from different departments, e.g., systems engineering students (Alvarez-Dionisi et al., 2019), medical students (Paranjape et al., 2019), and students with non-engineering backgrounds (Lin et al., 2021). AI is one of the most important subjects for medical students and related medical staff (Kolachalama and Garg, 2018; Paranjape et al., 2019). In today’s world of AI tools and technologies that provide excellent learning opportunities for teachers and students, teachers must consider the meaningful use of technology to teach rather in contrast to the meaningless use of technology (Zawacki-Richter et al., 2019). Because of the complexity of machine learning courses, there is a need to provide AI courses for non-information engineering undergraduates at an appropriate level of study. For example, Kong et al. (2021) indicated that AI literacy courses can promote students’ understanding of AI concepts in terms of self-perceived AI literacy and AI empowerment. Lindqwister et al. (2021) designed an AI tool in radiology (AI-RADS) course intended to provide high levels of student satisfaction and interest and to promote students’ understanding of AI concepts. Moreover, some courses, such as chemistry (Chen and Li, 2021) and English (Liu et al., 2021), use AI technologies and systems to assist with learning. However, at this stage of the AI curriculum development process, there is little research that can be used as a basis to implement an AI curriculum that is appropriate to the learning level of students or to teach such a curriculum to novices or non-information engineering undergraduates. An AI course, thus, has to be designed for novices or non-information engineering undergraduates.

## Method

### Research Participants

**FIGURE 1:**
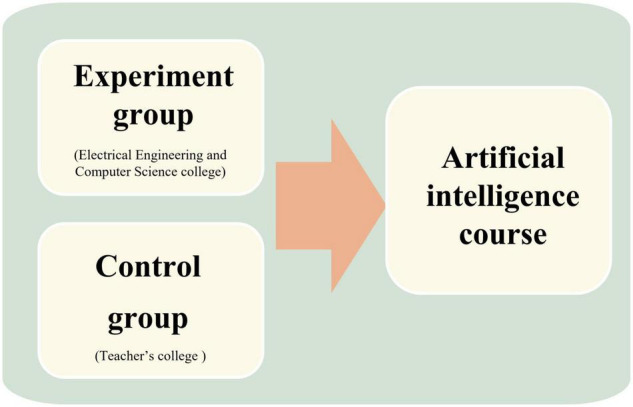
Research participants in the artificial intelligence (AI) course.

The participants of the study are shown in Figure 1. Convenience sampling was conducted to recruit the Science (Sci)-student and Humanities (Hum)-student groups. The Sci-student group was recruited from second-year electrical engineering students and the College of Computer Science at a national university. The Hum-student group was recruited from first-year students attending the College of Education at a national university. The difference between the Sci-student group and the Hum-student group was the depth and breadth of programming subjects studied in the different departments. However, both groups of students had basic programming experience but did not have a relevant AI learning experience.

### Research Process

**FIGURE 2:**
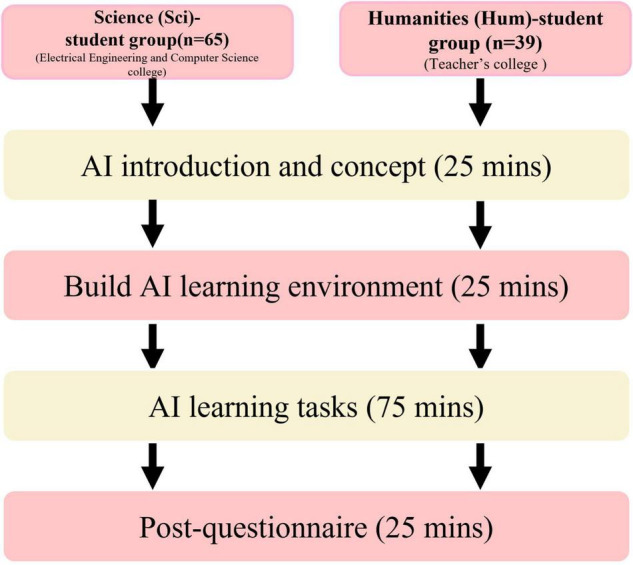
AI course research process.

The research process of the study is shown in Figure 2. Our teaching tool uses a self-built visual AI platform to deliver a 3-h AI course. The self-built visual AI platform provides a visual interface to reduce the difficulty of learning AI subjects for students. The course content included an introduction to AI and building an AI learning environment, a practical exercise on AI learning tasks, and completion of a questionnaire. In the first stage, both groups of students recruited from different national universities were given an introduction to AI and basic AI knowledge and concepts. In the second stage, the students built an AI platform environment and practiced on the AI platform to further develop their learning of AI. In the third stage, both groups of students had practical exercises on AI, and we carried out the AI learning task in the AI platform’s learning task 1 RGB-to-HSL. In the AI learning task stage, all the students followed the following learning steps: (1) inputting data sources from a computer, (2) adding new layers to the model and adjusting the relevant values, (3) modifying the detailed attribute fields in the new layers, (4) deciding on the final type of task to be performed, and (5) downloading the code of the model they created and trained. Finally, a questionnaire was distributed to the students with their consent, but the students were free to refuse to participate or not complete the survey. The Sci-student group comprised 65 students (55 men, 10 women), and the Hum-student group comprised 39 students (20 men; 19 women), and a total of 104 valid responses were obtained.

### Research Instrument

In this study, all questionnaires were based on a five-point Likert scale ranging from 1 to 5 (1 = completely disagree to 5 = completely agree). The reliability and validity of the research instruments were examined to ensure the reliability and validity of the questionnaires. A cognitive style questionnaire was used to understand the students’ cognitive tendencies toward learning. A computational thinking questionnaire was used to understand the computational thinking skills the students use when they study AI. To understand the differences in students’ cognitive style and computational thinking ability, questionnaires were used to measure students’ cognitive style and computational thinking ability. The Cognitive Style Questionnaire was modified from a study by Cools and Van den Broeck (2007), with 14 questionnaire items. The three variables included to measure cognitive style were creative style, planning style, and knowing style. The total Cronbach’s alpha value was 0.9. The reliability of the cognitive style variables was as follows: creative style (Cronbach’s alpha = 0.82), planning style (Cronbach’s alpha = 0.82), and knowing style (Cronbach’s alpha = 0.85). The validity of the questionnaire was examined by the Kaiser-Mayer-Olkin (KMO,0.86) and Bartlett’s (723.55, *p* < 0.001) tests. The total accumulated explanatory variance of these tests was 64.02%. The Computational Thinking Scale for Computer Literacy was modified from a study conducted by Tsai et al. (2021), with 19 questionnaire items. The five variables used to measure cognitive style included abstraction, decomposition, evaluation, algorithmic thinking, and generalization. The total Cronbach’s alpha value was 0.91. The reliability of the computational thinking variables was as follows: abstraction (Cronbach’s alpha = 0.86), decomposition (Cronbach’s alpha = 0.85), evaluation (Cronbach’s alpha = 0.78), algorithmic thinking (Cronbach’s alpha = 0.7), and generalization (Cronbach’s alpha = 0.72). The validity of the questionnaire was examined by the Kaiser-Mayer-Olkin (KMO,0.83) and Bartlett’s (1141.261, *p* < 0.001) tests. The total accumulated explanatory variance of these tests was 64.77%.

### Data Analysis

In this study, data analysis was performed with the SPSS software to analyze the differences in cognitive style and computational thinking in the Sci-student and Hum-student groups. An independent sample *t*-test was conducted to analyze the data from the two groups using the different groups as independent variables and cognitive style and computational thinking as dependent variables. The independent sample was examined for normative distribution, homogeneity of variance, and independent events. When these conditions were not met by independent sample testing, the Mann-Whitney *U*-test was conducted to analyze the data.

The values of the normality distributions of both groups are explained in Table 1. The Kolmogorov-Smirnova normality assumption is used for Sci-student groups with more than 50 participants, and the Shapiro-Wilk statistical normality assumption is used for Hum-student groups with lower than 50 participants. Significance (*p* > 0.05) means that the variable passed (Abstraction, Algorithmic Thinking) the normality distribution; therefore, conversely, significance of *p* < 0.05 means that the variable did not pass the normality distribution. For the non-normal distribution group, the Mann-Whitney *U*-test was conducted to examine any between-group differences.

**TABLE 1:** Verification of normal distribution values*.

Variable	Sci-student group (*n* = 65) Kolmogorov-Smirnova		Hum-student group (*n* = 39) Shapiro-Wilk	
	df	Sig	df	Sig
Creative style	65	0.00	39	0.00
Planning style	65	0.03	39	0.04
Knowing style	65	0.01	39	0.02
Abstraction	65	0.04	39	**0.06**
Decomposition	65	0.00	39	0.02
Evaluation	65	0.03	39	0.00
Algorithmic Thinking	65	0.00	39	**0.09**
Generalization	65	0.00	39	0.01

*Bold values indicate p-values > 0.05, which means that the variable passed the normality distribution.*

The values for the homogeneity of the two sets of variables are shown in Table 2. There were no significant differences in the variables, which means that the two groups were homogeneous.

**TABLE 2:** Verification of homogeneity test of variance*.

Variable	Levene statistic	Sig
Creative style	0.00	0.95
Planning style	0.82	0.37
Knowing style	0.35	0.56
Abstraction	0.01	0.94
Decomposition	0.33	0.57
Evaluation	1.16	0.28
Algorithmic Thinking	0.00	0.98
Generalization	0.37	0.55

*p < 0.05.

## Results and Discussion

Between-group differences in cognitive style and computational thinking were observed in the Sci-student and the Hum-student groups after the implementation of the AI course. The differences in cognitive style and computational thinking between the two groups are explained in Tables 3, 4. The Mann-Whitney *U*-test was conducted to analyze the data in terms of cognitive style and computational thinking skills.

**TABLE 3:** Analysis of the between-group differences in cognitive style*.

Variable	Sci-student group (*n* = 65)		Hum-student group (*n* = 39)		Mann-Whitney U	*p*
	Mean	SD	Mean	SD		
Creative style	3.78	0.63	3.78	0.58	1234.0	0.81
Planning style	3.87	0.66	3.77	0.60	1162.5	0.47
Knowing style	3.72	0.64	3.65	0.64	1209.5	0.69

*p < 0.05.

**TABLE 4:** Analysis of the between-group differences in computational thinking ability*.

Variable	Sci-student group (*n* = 65)		Hum-student group (*n* = 39)		Mann-Whitney U	*p*
	Mean	SD	Mean	SD		
Abstraction	3.61	0.78	3.69	0.80	1175.5	0.53
Decomposition	3.78	0.78	3.72	0.89	1212.5	0.70
Evaluation	4.05	0.54	4.04	0.51	1228.5	0.78
Algorithmic Thinking	3.53	0.69	3.76	0.58	1032.5	0.10
Generalization	3.67	0.61	3.76	0.60	1156	0.44

*p < 0.05.

The findings of this study showed that the students in the two groups did not have significant differences in terms of creative style (Mann-Whitney *U* = 1,234, *p* > 0.05), planning style (Mann-Whitney *U* = 1,162.5, *p* > 0.05), and knowing style (Mann-Whitney *U* = 1,209.5, *p* > 0.05). No significant between-group differences are found in terms of cognitive style after the post-test, as shown in Table 3. The findings were not surprising, since cognitive styles have stable learning characteristics (Bouckenooghe et al., 2016), and students are unlikely to change their cognitive style in a short period of time after taking an AI course. Another possible explanation for this finding is that both groups of students had similar cognitive styles before the experiment. After the experiment, both groups still had similar cognitive styles. The findings of this study also imply that the cognitive styles of students from different disciplines are an important issue in this field. In particular, Billington et al. (2007) indicated that among students’ systemic and empathic characteristics, science students have higher systemic characteristics, and humanities students have higher empathic characteristics. Furthermore, Katai (2020) indicated that in two groups of students (humanities and science students), differences in academic achievement were reduced as their learning progressed in a suitable e-learning environment. In this study, understanding the cognitive styles of students in different subjects helped teachers design appropriate AI courses that incorporate, e.g., the use of visual tools to reduce learning difficulties and increase student engagement. An AI course was implemented for a relatively short period of time. Therefore, it was difficult to assess the changes in students’ cognitive style when using visual programming. However, according to computational thinking research, teachers should help students understand the concept of computational thinking in a meaningful way by explaining the programming process and using real-life examples to explain computational thinking (Voogt et al., 2015). Similarly, teachers can explain AI with specific examples and use appropriate teaching tools in AI courses. This approach may influence students’ cognitive styles by changing their computational thinking.

The study findings show that the university students in the two groups did not have significant differences in terms of abstraction (Mann-Whitney *U* = 1.175.5, *p* > 0.05), decomposition (Mann-Whitney *U* = 1,212.5, *p* > 0.05), algorithmic thinking (Mann-Whitney *U* = 1,228.5, *p* > 0.05), evaluation (Mann-Whitney *U* = 1,032.5, *p* > 0.05), and generalization (Mann-Whitney *U* = 1,156, *p* > 0.05) (Table 4). This study was limited by the short duration of the experiment and the fact that computational thinking is a high-level skill that is difficult to develop in a short period of time. To develop students’ computational thinking skills, it is necessary to design and adjust curricula to suit the learning level of students in different disciplines (Katai, 2020). However, it is important to note that the results of the study did show that the Hum-student group had higher scores in abstraction (Sci-student group = 3.61, Hum-student group = 3.69), algorithmic thinking (Sci-student group = 3.53, Hum-student group = 3.76), and generalization (Sci-student group = 3.67, Hum-student group = 3.76) than the Sci-student group. This may indicate that the Hum-student group was more perceptually influenced in the learning process during the AI course in terms of visual programming. In addition, Karalar and Alpaslan (2021) indicated that students with more experience in programming have higher computational thinking skills than those with no programming experience. The Sci-student group had more programming experience, and the Hum-student group had less, but the findings were not consistent with those of previous studies (Karalar and Alpaslan, 2021). This was because the students manipulated the visual programming block learning process (e.g., modeling), which made it possible for them to repeat computational thinking patterns that would enhance their computational thinking skills. For example, machine learning requires the construction of models, and students learn computational thinking skills by sequencing the types of models (algorithms) and designing model steps (modularization). That is to say, the learning benefits of using visual programming blocks (e.g., ease of use) can make it possible for two different groups of university students to acquire similar computational thinking skills. All students spent the same time learning about machine learning, and visual programming reduced the difficulty of learning machine learning and helped the students to effectively understand the learning process.

## Conclusion

This study examines the differences in cognitive style and computational thinking during the implementation of AI courses among a sample of university students from different departments. AI courses present complex and difficult learning challenges. An AI platform with visual interfaces plays a key role to help students learn AI easily. However, teaching a one-size-fits-all approach for content delivery may increase failure and dropout rates. A student-centered AI content design and use of appropriate teaching tools and teaching strategies (e.g., visualized programming) can reduce learning difficulties and expand the learning of AI subjects in students with different learning backgrounds. As proposed in this study, the implementation of visual programming blocks in the AI curriculum helped students and students from different learning backgrounds learn about AI. There were no significant between-group differences in cognitive style and computational thinking among the university students after taking the AI course. To help students from different learning backgrounds learn AI, the use of appropriate teaching tools and teachers who can explain programming thinking may help students better understand AI. In addition, a visual programming learning environment may be a potentially important technology in the field of AI to help all students easily learn about AI. This study discusses a means by which all students can learn about AI, allowing them to learn AI concepts without the constraints of programming syntax, questions, and practical learning tasks.

## Limitation and Future Studies

This study was limited by the lack of pre-testing to examine students’ cognitive style and computational thinking abilities, which means that we did not know the original learning status of the students. and it was also limited by short implementation time. In this study, the survey was only given to university students from two different departments, which made it impossible to learn about the learning situation of general university students. In general, the majority of university students in the Electrical Engineering and Computer Science College are interested in and major in computer science. The majority of university students in the College of Education had a wide range of learning interests and a preference for studying literary subjects. However, to understand the learning situation of different university students in terms of the AI curriculum, the AI technology could be used to monitor and collect data on students’ learning history to provide a better understanding of changes in the learning situation of three different groups of university students. Future research could consider a systematic survey of AI technology and the use of general university student surveys to compare the learning of AI among three groups. Finally, the lack of a long-term evaluation of the effectiveness of the course limits the understanding of its impact. However, it is important to note that different learners have different ways of processing information (i.e., different cognitive styles) and develop different computational thinking skills during an AI course. It would be interesting to discover what learning processes change the cognitive style and computational thinking skills of successful AI learners after a long period of study, because this information is essential for instructional designers and educators, so it would be useful to conduct research on changes in learning processes around cognitive style and computational thinking skills. Future research should examine the learning outcomes of using AI courses over time and evaluate the learning benefits of AI as they relate to different aspects of learning (e.g., self-regulated learning).

## Data Availability Statement

The original contributions presented in the study are included in the article/supplementary material, further inquiries can be directed to the corresponding author.

## Ethics Statement

Ethical review and approval was not required for the study on human participants in accordance with the local legislation and institutional requirements. Written informed consent from the participants’ legal guardian was not required to participate in this study in accordance with the national legislation and the institutional requirements. Written informed consent was obtained from the individuals for the publication of any potentially identifiable images or data included in this article.

## Author Contributions

C-JW was responsible for analyzing and interpreting the data. J-HC was in charge of designing the research framework, collecting and interpreting the data, and revising the article. P-SC was responsible for collecting the data and revising the article. P-HW was in charge of designing the research framework and collecting the data. H-XZ was responsible for the data analysis, the design of the research framework, and wrote the first draft of the manuscript. J-HC, C-JW, and H-XZ did the overall design of the study. J-HC and C-JW reviewed, edited, and supervised the article. All authors have read and approved the final version of the manuscript, designed the questionnaire, collected the data, and wrote sections detailing the sample of students and data collection, made a substantial, direct, and intellectual contribution to the study, and agreed to the published final manuscript.

## Conflict of Interest

The authors declare that the research was conducted in the absence of any commercial or financial relationships that could be construed as a potential conflict of interest.

## Publisher’s Note

All claims expressed in this article are solely those of the authors and do not necessarily represent those of their affiliated organizations, or those of the publisher, the editors and the reviewers. Any product that may be evaluated in this article, or claim that may be made by its manufacturer, is not guaranteed or endorsed by the publisher.
